# Three-Dimensional Modeling of Avascular Tumor Growth in Both Static and Dynamic Culture Platforms

**DOI:** 10.3390/mi10090580

**Published:** 2019-08-31

**Authors:** Ali Taghibakhshi, Maryam Barisam, Mohammad Said Saidi, Navid Kashaninejad, Nam-Trung Nguyen

**Affiliations:** 1 Department of Mechanical Engineering, Sharif University of Technology, Tehran 11155, Iran; 2 Queensland Micro- and Nanotechnology Centre, Griffith University, Nathan Campus, 170 Kessels Road, Brisbane, QLD 4111, Australia

**Keywords:** Tumor modeling, Microfluidic U-shaped, Tumor growth rate, Tumor kinetics, Cell culture numerical simulation

## Abstract

Microfluidic cell culture platforms are ideal candidates for modeling the native tumor microenvironment because they can precisely reconstruct in vivo cellular behavior. Moreover, mathematical modeling of tumor growth can pave the way toward description and prediction of growth pattern as well as improving cancer treatment. In this study, a modified mathematical model based on concentration distribution is applied to tumor growth in both conventional static culture and dynamic microfluidic cell culture systems. Apoptosis and necrosis mechanisms are considered as the main inhibitory factors in the model, while tumor growth rate and nutrient consumption rate are modified in both quiescent and proliferative zones. We show that such modification can better predict the experimental results of tumor growth reported in the literature. Using numerical simulations, the effects of the concentrations of the nutrients as well as the initial tumor radius on the tumor growth are investigated and discussed. Furthermore, tumor growth is simulated by taking into account the dynamic perfusion into the proposed model. Subsequently, tumor growth kinetics in a three-dimensional (3D) microfluidic device containing a U-shaped barrier is numerically studied. For this case, the effect of the flow rate of culture medium on tumor growth is investigated as well. Finally, to evaluate the impact of the trap geometry on the tumor growth, a comparison is made between the tumor growth kinetics in two frequently used traps in microfluidic cell culture systems, i.e., the U-shaped barrier and microwell structures. The proposed model can provide insight into better predicting the growth and development of avascular tumor in both static and dynamic cell culture platforms.

## 1. Introduction

Cancer has always been a significant threat to human beings. The global rate of deaths associated with this fatal disease is alarming. In 2016, 17.2 million people were estimated to live with cancer, and cancer accounted for 8.9 million deaths worldwide [1], which elucidates that it is a critical issue to be concerned about. Therefore, evaluating the characteristics of tumors and their growth process is of paramount importance and is useful for obtaining successful treatment.

Avascular growth phase is the common phase between both benign and malignant tumors and can be better experimentally simulated using in vitro platforms. In this phase, the growth rate is limited to diffusion and consumption of the nutrients [2]. Studies in avascular tumor growth by means of diffusion have been extensively conducted over the past decades, and many models have been presented. In early models, the process of the diffusion of the dissolved substance through tissues as well as their consumption by metabolic processes was studied [3]. Subsequently, a few experimental studies—both in vitro and in vivo—focused on radiotherapy and the effects of radiation on the tumor growth [4,5]. Thomlinson and Gray [6] extensively examined the effects of radiations on hypoxic tumor cells, which were proved to be radio-sensitive and less vulnerable to X- and γ-radiations.

In 1966, Burton [7] established a new mathematical model which examined oxygen distribution inside a tumor spheroid. The model was capable of determining the radius of the central zone of a tumor, and the growth curve obtained by the model could fit a well-known mathematical function, the Gompertzian function. Thereafter, Greenspan [8] presented a model by considering the living cell adhesion, which results in an inward pressure in spheroids. The author also assumed the disintegration of necrotic cellular debris into permeable, simpler compounds whose volume loss was compensated by some cells that were pushed inward due to the surface tension and adhesion. The authors concluded that the inhibitory chemicals were produced in the necrotic core. These chemicals could retard the tumor growth by preventing cells from mitosis, without causing the death of cells. Depending on whether the inhibitory chemicals were produced due to the insufficient nutrient supply and a necrosis product or by the metabolic process of living cells, the model would predict two utterly different growth patterns. 

McElwain and Morris [9] developed a new mathematical model for tumor growth. The tumor passes through three different phases according to their model. During the first phase, cells are supplied with a sufficient amount of nutrient (oxygen) and cell mitosis proceeds ordinarily. In the second phase, the growth rate decreases until the nutrient reaches its critical value, and in the third phase, the growth rate further decreases, and the cell mitosis rate is in balance with tumor volume loss. The authors adopted in their model Greenspan’s assumption for cell loss mechanism—the disintegration of living cells after being forced inward by surface tension and adhesion. In a later study [10], the same group improved their model by considering apoptosis as a cell loss mechanism in the tumor. Using the general approach developed in their previous paper, they applied the apoptosis mechanism to all the cells except those in the necrotic core. The authors concluded that the tumor might reach a dormant state in the second phase, as the apoptosis rate may balance the cell mitosis rate; and otherwise, the growth proceeds to the third phase. Inspired by McElwain and Morris’s model, Lakshminarayanan et al. developed a model for tumor growth based on nutrient diffusion as well as apoptosis and necrosis as cell loss mechanisms [11]. 

Casciari et al. introduced a model for the growth of multicellular tumor spheroids based on oxygen and glucose concentrations, as well as the culture medium pH [12]. The authors investigated the shedding of mitotic cells from the surface of the tumor spheroid as a cell loss mechanism. The team also defined a maximum growth rate fraction, which was a function of glucose and oxygen concentrations as well as the culture medium pH, whose multiplication in the tumor volume would be the growth rate. They also conducted some experiments regarding culturing and observing the tumor growth and utilized their experimental data to verify their model [13]. In the later experiments, Freyer and Sutherland [14] cultured multicellular spheroids in dishes with various oxygen and glucose concentrations for more than three weeks. Long enough culture time in their study allowed the tumors to pass through different stages of growth; accordingly, their experimental data are appropriate for evaluating models of tumor growth. 

As a precise simulation of in vitro process, mathematical models of tumor growth can be employed to investigate tumor growth procedure in microfluidic devices. In fact, microfluidic approaches are widely used for examining a diverse range of phenomena [15,16,17,18], and using microfluidic devices is a common approach to study different characteristics of cancerous tumors [19,20,21,22,23,24,25]. Hu and Li [21] simulated tumor growth in a microchannel bioreactor. They used Casciari’s model as well as Navier-Stokes equations to investigate the growth of the tumor exposed to the culture medium flow. By repeating the simulations for different inlet velocity of the culture medium, the authors concluded that the tumors grow faster under high inflow velocity, because of the fast nutrient supply to the tumor. They monitored tumor growth for fewer than 80 h. Given the nutrient concentrations and their simulation time, it can be concluded that the tumors in their simulations do not develop the necrotic zone.

Herein, we modify Lakshminarayanan’s model [11] for better agreement with experimental data [14]. Even though our modified model is still based on the nutrient diffusion and considers apoptosis as well as necrosis as the main cell loss mechanisms, the tumor growth kinetics is modified in such a way that the nutrient consumption rate of the cells in the quiescent zone is half of that in the proliferative zone, according to Jiang et al. [26]. With the employed modifications, the proposed modified model can better predict the experimental results in the literature [14]. We further investigate the effects of the concentrations of the essential nutrients and the initial spheroid radius on the tumor growth. As the next step, we include the effect of dynamic perfusion of the culture medium into the model and examine the tumor growth in microfluidic devices. Despite previous studies which simulated the tumor growth for a short duration, we simulate the tumor growth in a microfluidic device containing U-shaped barrier for 300 h. This approach enables time-dependent investigation of the different stages of the tumor growth and quantifies dynamic necrotic as well as quiescent zones in the tumor. Moreover, we evaluate the effect of the flow velocity of the culture medium on the tumor growth rate. Finally, to investigate how the geometry of the trap may affect the spheroidal tumor growth, we simulate the tumor behavior in a microchip with a microwell trap and compare the results to those obtained in the U-shaped barrier.

## 2. Materials and Methods 

### 2.1. Tumor Growth Model

**Figure 1:**
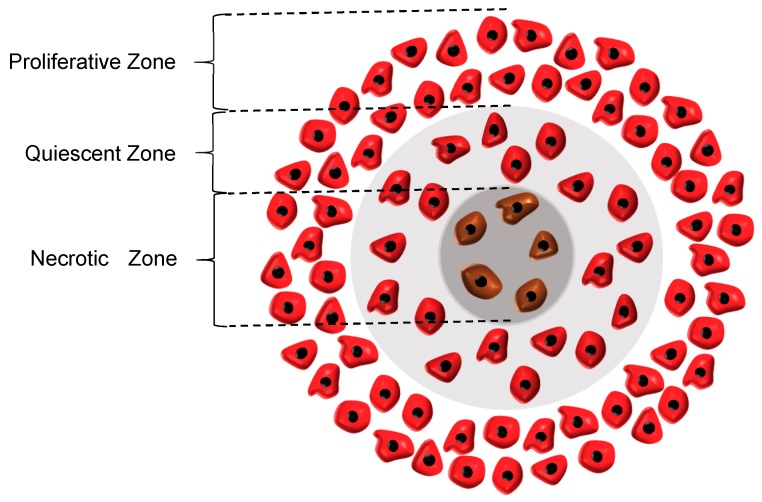
Three main regions in a tumor. Proliferative zone, nutrients concentrations are above their critical value; Quiescent zone, the concentration of one of the nutrients fall below its critical value; Necrotic zone, nutrients concentrations are below their critical value.

The equations used for the tumor growth are based on the analytical model of Lakshminarayanan et al. [11]; however, some reasonable modifications have been applied to the proposed model so as to make it more compatible with the experimental data. In this model, two main cell-loss mechanisms are considered, namely apoptosis and necrosis. Moreover, the growth of the tumor is directly affected by the concentration distributions of oxygen and glucose, which are the two essential components for cancerous tumors to thrive. As such, the whole tumor volume is divided into three main regions, i.e., proliferative, quiescent and necrotic zones. In the proliferative zone, adequate nutrients are available for all the cells. Accordingly, the cells grow at the maximum rate in this zone. As the tumor grows, however, the quiescent zone appears and the concentration of one of the nutrients falls off below its critical value. Hence, according to Jiang et al. [26], the rate of cell growth in this zone declines to half of its initial rate. Although the cells in the quiescent zone are not dead yet and still take up some nutrients, they are regarded as non-dividing cells. Subsequently, the necrotic region appears when the concentrations of both oxygen and glucose are below their critical values, causing all the cells in this zone to die. As a result, the growth rate in this zone is zero and the necrosis mechanism retards the tumor growth. It also should be noted that in the whole tumor volume except the necrotic area, the apoptosis mechanism acts as an inhibitory factor against the tumor growth. The three main regions in a tumor are graphically displayed in Figure 1.

#### 2.1.1. Nutrient Concentrations

The concentrations of nutrients can be obtained using the general form of convection-diffusion-reaction equation:

(1) $$\partial C/\partial t+\vec{V}\cdot \vec{\nabla }C=\vec{\nabla }.\left(D\vec{\nabla }C\right)-R$$

where C, D and R are the concentration, diffusion coefficient and reaction rate of a nutrient, respectively. Since the velocity of nutrients inside the tumor is negligible, a simplified yet logical assumption is to neglect the convection term in the left-hand side of Equation (1). In addition, by considering a constant value for the diffusion coefficient, Equation (1) can be simplified for the three regions mentioned above.

Equations (2)–(4) are related to the nutrient consumption in proliferative, quiescent and necrotic zones, respectively.

(2) $$\partial C_{i}/\partial t=D_{i}/r^{2}\partial /\partial r\left(r^{2}\partial C_{i}/\partial t\right)-\left(V_{i}C_{i}/K_{i}+C_{i}\right)$$

(3) $$\partial C_{i}/\partial t=D_{i}/r^{2}\partial /\partial r\left(r^{2}\partial C_{i}/\partial t\right)-1/2\left(V_{i}C_{i}/K_{i}+C_{i}\right)$$

(4) $$\partial C_{i}/\partial t=D_{i}/r^{2}\partial /\partial r\left(r^{2}\partial C_{i}/\partial t\right)$$

In the above equations, *r* is the radial position in the tumor; the last term on the right-hand side of Equation (2) is the Michaelis-Menten reaction term; *i* = 1, 2 identifies glucose and oxygen, respectively; V is the maximum reaction rate of the nutrients, and *K* is the Michaelis constant of the nutrients. Moreover, in contrast to the Lakshminarayanan’s model [11], where the authors considered no nutrient consumption in the quiescent zone, here we consider the fact that the consumption rate of nutrients is half in the quiescent zone compare to that in the proliferative zone [26]. In addition, due to the lack of sufficient nutrients, the cells in the quiescent region are assumed not to proliferate.

#### 2.1.2. Growth Equation

Based on what was mentioned in the model definition, during the growth process, tumor passes through three phases. The tumor is in the first phase of its growth until the quiescent zone comes into existence. The second phase begins with the appearance of the quiescent zone and lasts until the necrotic zone appears. The onset of the necrotic zone is the beginning of the third phase: the last phase of the growth. The rate of volumetric change of tumor follows different patterns in the three phases. The general growth equation with radial growth assumption is as follows:

(5) $$dV/dt=A\left(V-V_{q}\right)-\left(A_{p}\left(V-V_{n}\right)\right)-\left(A_{n}V_{n}\right)$$

where V, $V_{q}$ and $V_{n}$ are the volume of the spheroidal tumor, quiescent and necrotic zones, respectively. Additionally, A, $A_{p}$ and $A_{n}$ are respectively the proliferative, apoptosis and necrosis constants. It is noteworthy that $V_{q}$ is zero during the first phase. Moreover, since the necrotic core appears in the third phase, $V_{n}\;$ is zero in the first two phases. Using the above equations, the tumor radius can be determined as a function of time.

#### 2.1.3. Initial and Boundary Conditions

As an initial condition, we assume that at the beginning of the growth, the concentrations of both nutrients are equal to that of the culture medium:

(6) $$C_{i}=C_{i\infty }\;\mathrm{at}\;t=0\;\mathrm{and}\;\mathrm{all}\;r$$

In the model, the tumor spheroid is exposed to an unlimited, static culture medium. Thus, as a boundary condition, the concentrations of both nutrients are equal to that of the culture medium at the tumor surface:

(7) $$C_{i}=C_{i\infty }\;\mathrm{at}\;r=R\;\mathrm{and}\;\mathrm{all}\;t\;$$

#### 2.1.4. Applying the Modified Model to Tumor Spheroid in Microfluidic Devices

By assuming that the tumor grows radially [21], the model introduced above can be employed for simulating the growth of the tumor in microfluidic devices. Additionally, for modeling tumor growth in microfluidic devices, continuity and momentum equations which are related to the nutrient flow through the microchannel must be considered:

(8) $$\vec{\nabla .}\vec{V}=0$$

(9) $$\rho (\vec{\nabla .}\vec{V})\vec{V}=-\vec{\nabla }p+\mu^{2}\vec{V}$$

where *ρ*, *μ*, $\vec{V}$ and $\vec{\nabla }p$ are the fluid density and viscosity, velocity vector, and pressure gradient, respectively.

As it is further discussed in Section 4, we assume steady-state culture medium flow for the tumor growth. Also, for the diffusion of the nutrients through the culture medium, since there is no consumption of the nutrients outside the tumor spheroid, Equation (1) is simplified as follows:

(10) $$\vec{V}\cdot \vec{\nabla }C=D\nabla^{2}C$$

Accordingly, Equations (8)–(10) are coupled and solved numerically in order to find the nutrient concentrations in the culture medium. The parameters of the equations are listed in Table 1.

### 2.2. Geometry

**Figure 2:**
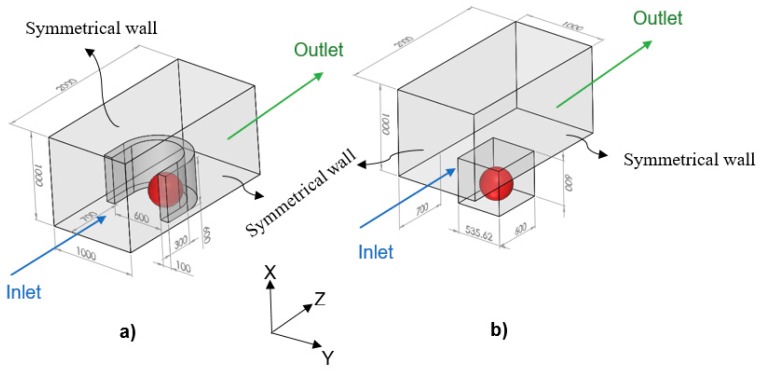
Tumor spheroid (the objects in red) in a microchannel containing: (**a**) A U-shaped barrier; (**b**) A microwell trap. The dimensions are in micrometer.

In the model, since the tumor is exposed to the static, unlimited culture medium and nutrient concentrations at the tumor surface are constant, the distributions of the nutrient concentrations are spherically symmetric. Therefore, the nutrient concentrations change only in the radial direction, and accordingly, we consider the tumor geometry an axially symmetric semicircle. Since the spherical symmetry is assumed, there is no difference between the results obtained by a sphere or a semicircle as the tumor geometry; however, considering the tumor as a semicircle will be more numerically efficient (this issue will be further discussed in the numerical method section). Inspired by Barisam et al. [27], the microfluidic devices studied in this research contain traps in the form of a microwell or a U-shaped barrier. Due to the symmetry condition of the side surfaces of the microchannels, only one trap has been considered in the geometry of each one. Also, all of the surfaces in both types of microchannels are impermeable to the nutrients. As shown in Figure 2, the traps have the same length, height and cross-section area. Moreover, the centers of both types of traps superpose the center of the lower surface of the microchannel.

### 2.3. Numerical Method

The first five equations, which are related to the growth and the concentration distributions, are solved numerically for achieving an approximate solution. As mentioned in the geometry section, for the condition of static, unlimited culture medium, the tumor is assumed as an axially symmetric semicircle, the revolution of which around its axis will be the actual 3D tumor in the model. Since the tumor assumes the shape of a hemisphere, it requires much fewer computational cells for a numerical solution than a sphere and can be solved more easily, without making any difference in the results.

Tessellation method is utilized to mesh the tumor exposed to the unlimited, static culture medium, and unstructured triangular grids with initial sizes between 9.71 $\times \;10^{-4}\;\mu m$ up to 4.85 $\times \;10^{-1}\;\mu m$ are generated. Finite element approach is used with a residual value less than $10^{-3}$ for growth as well as diffusion-reaction equations. For the tumor placed in a microfluidic device, unstructured tetrahedral grids are generated which are between 4 $\times \;10^{-1}\;\mu m$ up to 1.2 μm for the tumor domain and between 5.06$\;\times 10^{1}\;\mu m$ up to 1.64$\;\times 10^{2}\;\mu m$ for the microchannel zone. In this case, mesh refinement has been employed to the boundaries as well as interface surfaces. Convergence criteria to solve the equations of continuity, momentum, concentration distribution, and tumor growth are selected as residual values less than $10^{-6}$, $10^{-6}$, $10^{-3}$, $10^{-3}$, respectively.

For a specific flow rate and tumor radius, the flow equations (i.e., Equations (8) and (9)) along with the convection-diffusion equation (Equation (10)) need to be solved only once in order to obtain the distributions of the nutrient concentration in the culture medium. The time required for the concentrations of the nutrients to reach a steady-state, both inside the tumor and in the microchannel, is far much shorter than the growth time of the cells. Once the nutrients concentrations are determined, for a given tumor radius, the growth rate of the tumor is calculated using the growth equations (by calculating the volume occupied by the whole tumor, quiescent, and necrotic zones and employing Equation (5). After 20 h, the tumor radius is updated using the growth rate obtained. The same procedure is repeated for the new tumor radius.

It is noteworthy that the conditions and the distribution of the nutrients within a tumor in the microfluidic device is asymmetric. However, this cannot affect the accuracy of applying the present model to tumor growth in a microfluid device because in the proposed model, the growth equation is simply influenced by the volume of different regions of the tumor, not by its geometrical distribution. 

### 2.4. Model Validation

**Figure 3:**
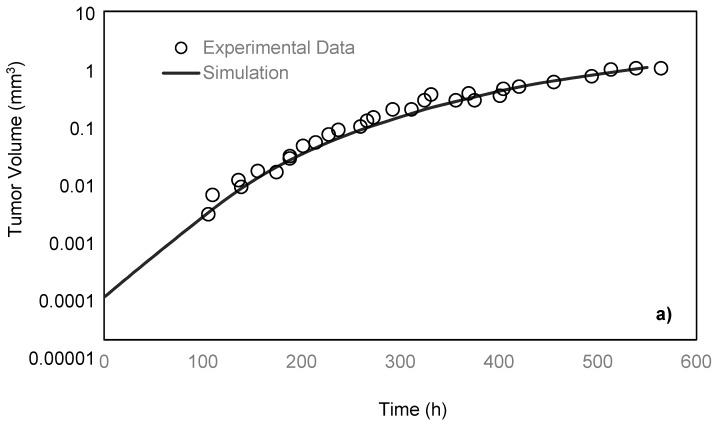

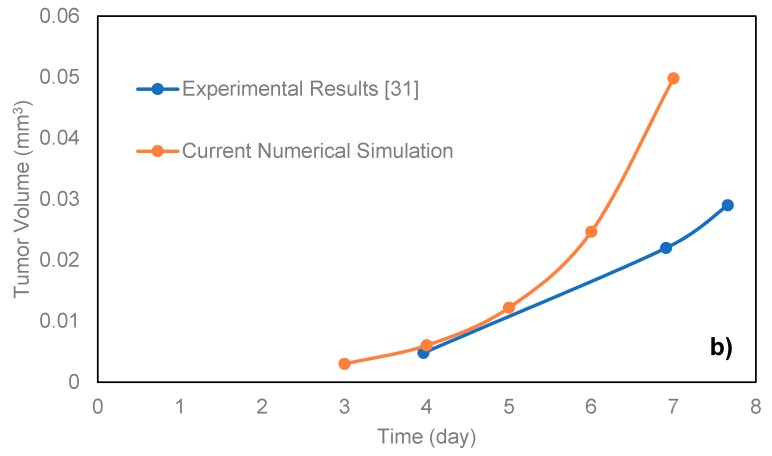
(**a**) Comparison of the tumor volumes from experiment data of the Sutherland and Freyer for EMT6/Ro spheroid [14] and simulation based on the proposed model. In both cases, the tumor is exposed to the unlimited static culture medium, and the glucose and oxygen concentrations in the culture medium are 0.8 mM and 0.28 mM, respectively. (**b**) Applying the model for HT-29 tumor growth in proliferative phase in a microfluidic device with a microwell trap proposed by Ziółkowska et al. [31]. To better validate the results, on-chip measurements of specific parameters of the HT-29 cell line was necessary. These parameters included the necrosis and apoptosis rates, the diffusion coefficient of the nutrients to the HT-29 tumor spheroids, as well as Michaelis-Menten parameters of the nutrients.

Freyer and Sutherland [14] cultured mouse mammary carcinoma spheroids in different concentrations of glucose and oxygen and studied how the nutrient concentrations affect the tumor size. To maintain a steady-state and constant-concentration medium, the authors replenished the spinner flask, in which the tumors were placed every 10–14 h. To measure the spheroid size, they measured two orthogonal diameters of the spheroids by means of an inverted microscope, which was fitted with a calibrated eyepiece reticule. After monitoring the tumors for 28 days, the team reported the growth rate of the tumor by obtaining the values of its volume over time. In the present paper, the simulation results are compared with their experimental data under 0.8 mM glucose and 0.28 mM oxygen medium concentrations. As previously discussed, a spherically symmetric tumor is exposed to the static, unlimited culture medium, and its growth kinematics has been monitored. It is assumed that the concentrations of the nutrients are constant in the culture medium since it is unlimited. The growth starts with a single cell, which will develop into a tumor spheroid containing proliferative, quiescent and necrotic zones [26]. Using the proposed model, the simulation curve is obtained, which is in a good agreement with the experimental data of the EMT6/Ro spheroid, compared to the previous studies (Figure 3a). 

For the comparison of the proposed model with microfluidic systems, the exact relevant values of various parameters should be experimentally measured. As on-chip measurements of such parameters in a microfluidic platform require integration of related biosensors with the chip, there are very limited numbers of studies in this field. Here, the model was compared with the data of the microfluidic platforms proposed by Ziółkowska et al. [31]. In that work, the authors used HT-29 human carcinoma cells cultured in a microfluidic system with microwell arrays in the form of multicellular spheroids with a cell density of 1.5 × 10^6^ cells/mL. The authors only provided the exact value of the proliferation rate constant of HT-29. Here, we applied the model for the proliferating phase of the HT-29 tumor spheroids in the dynamic microwells, Figure 3b. Although the general trend is achieved, there are some disagreements between the experimental and simulation results. This is directly due to the lack of knowledge about some necessary parameters of HT-29 cell line, such as the necrosis and apoptosis rates, the diffusion coefficient of the nutrients to the HT-29 tumor spheroids, as well as Michaelis-Menten parameters of the nutrients. The only known parameter was the proliferation rate constant, and the rest of the parameters were adopted from EMT6/Ro cell line. The better agreement could be achieved in future studies by replacing the exact parameters of HT-29.

## 3. Results and Discussion

In this section, for a spheroid placed in an unlimited, static culture medium, the effects of nutrient concentrations as well as the initial volume of the tumor on its growth rate are investigated. Moreover, the results of simulations of the tumor growth in a microchannel containing U-shaped barrier are shown and compared to those in a microchannel with a microwell.

### 3.1. Tumor Growth in Static, Unlimited Culture Medium

#### 3.1.1. Effect of Nutrient Concentration on Final Tumor Volume

**Figure 4:**
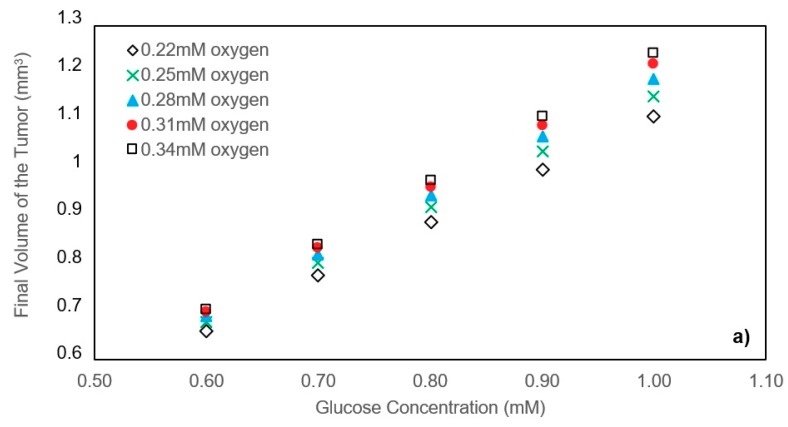

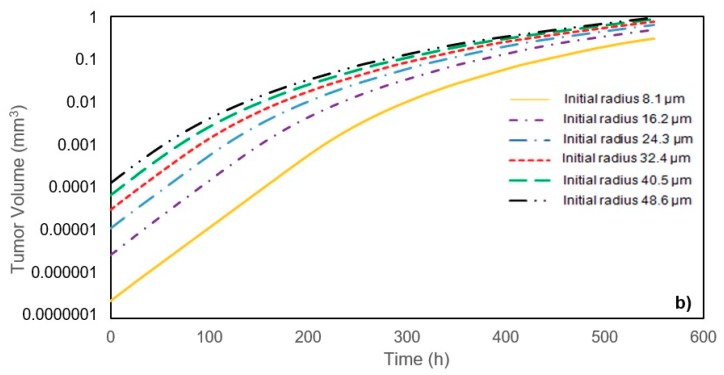
(**a**) Effect of nutrient concentrations on the tumor volume after 550 h. Tumors with 24.3 μm initial radius are exposed to unlimited static culture mediums varying in nutrient concentrations. (**b**) Effect of the initial tumor radius on the growth. All of the tumors are exposed to the unlimited static culture medium with 0.8 mM glucose and 0.28 mM oxygen concentration.

By changing the oxygen and glucose concentrations of about 0.28 mM and 0.8 mM, respectively, the effect of nutrients concentration on the final volume of the tumors is investigated for the case with good agreement between model and the experimental data. Figure 4a shows the volume of the tumors after 550 h in each state with oxygen concentration varying from 0.22 mM to 0.34 mM and glucose concentration varying from 0.6 mM to 1 mM. The initial radius of all the tumors is the same, and equal to 24.286 μm. It is apparent that higher nutrient concentrations will lead to a larger final volume of the tumor. This is mainly because higher nutrient concentrations retard the formation of the necrotic and quiescent zones. In addition, we observed that the effect of glucose concentration on the final volume is more significant than that of oxygen. The reason for such difference lies in the diffusion coefficients of glucose and oxygen as well as the consumption rate of these nutrients. Since oxygen molecules are much smaller than glucose ones, they can permeate more easily into the tumor spheroid. Furthermore, in the range of nutrient concentrations studied in this part, glucose concentration fell off to its critical value in the center of the tumor sooner than oxygen. Therefore, in these cases, the glucose is the determinative nutrient, and a change in glucose concentration has more impact on the final tumor volume than the same change in oxygen concentration.

**Figure 5:**
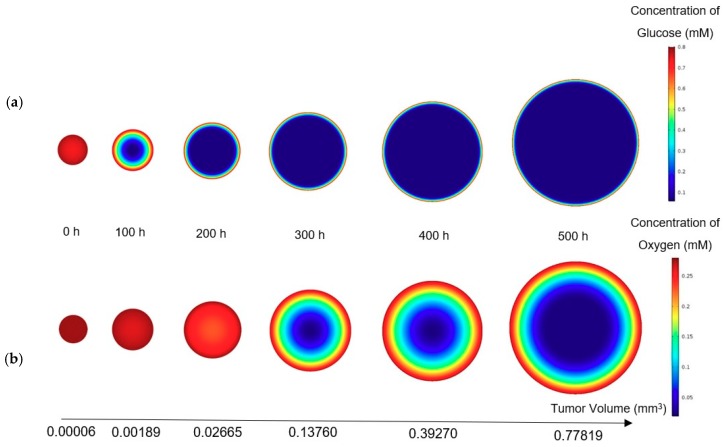

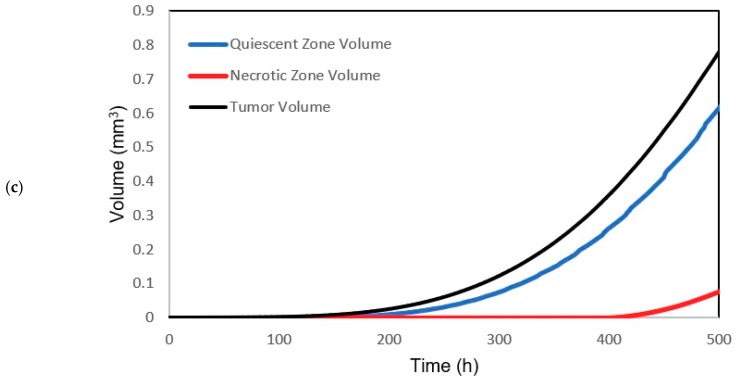
(**a**) Glucose; (**b**) Oxygen concentration during the growth of a tumor (concentrations of glucose and oxygen in the culture medium are constant versus time and they are respectively 0.8 mM and 0.28 mM). The initial radius of the tumor is 24.3 μm. (**c**) Volume distributions of the different regions of the tumor.

The concentrations of glucose and oxygen determine the volume occupied by the quiescent and necrotic zones, respectively. Moreover, the contours of the nutrient distribution during the growth are shown in Figure 5, where the concentration of glucose and oxygen are 0.8 mM and 0.28 mN, respectively. The ratio of the size of the sections is not real and is only shown to induce tumor growth. In fact, two sections with real size cannot be displayed side by side, because of their large difference. In contrast to the oxygen distribution, the concentration of glucose dropped significantly inside the tumor. In addition, the volume of the different zones in the tumor in the mentioned condition during tumor growth is shown against time in Figure 5c.

#### 3.1.2. Effect of Initial Tumor Radius on Tumor Growth

In an unlimited, static culture medium with the oxygen and glucose concentrations respectively equal to 0.24 mM and 0.6 mM, and by changing the initial tumor radius from 8.1 μm to 48.6 μm, the results in Figure 4b indicate that the initial tumor radius does not play a significant role in the final volume of the tumor. In tumors with smaller initial radius, the nutrients can diffuse and reach the core of the tumor easily, retarding the quiescent and necrosis zones. These zones, however, appear sooner in tumors with larger initial radius. Initially, because the spheroid diameter is small enough and the quiescent and necrosis areas have not yet formed, the growth rate in all cases is equal to the growth rate of the proliferating cells. At the end of the graphs, as the rate of proliferation and mortality approaches, the growth rates tend to zero. However, between the start and the end, they will experience different growth rates due to the lack of synchrony in the formation of quiescent and necrosis areas.

Accordingly, it can be concluded that the smaller the initial radius, the higher the tumor growth rate and vice versa. As it can be interpreted from the data in Figure 4b, tumors with different initial radii exposed the same nutrient concentrations will converge to the same final volume.

### 3.2. Tumor Growth in a Microchannel Containing U-shaped Barrier

When a tumor is placed in a perfusion-based microfluidic device, it is exposed to continuous culture medium flow, containing oxygen and glucose as diluted species. In this study, concentrations of oxygen and glucose are 0.28 mM and 0.8 mM, respectively. Since the tumor consumes the surrounding nutrients, the nutrient concentrations at the tumor surface are not the same as of the internal flow. Moreover, the shape of the barrier itself leads to the reduction of the nutrient concentrations in the tumor surface, which accelerates the formation of the quiescent and necrotic zones in the tumor [19,27]. Hence, as expected, the growth rate of the tumor placed in a continuous perfusion-based microfluidic device is slower compared to the case of static, unlimited culture medium. Tumor volume, as well as the volume of the quiescent and necrotic zones, are inspected during the growth time for three different inflow velocities. During the tumor growth, the maximum shear rate in the tumor among all the inlet velocities does not exceed 0.227 dyn/cm^2^. 

High flow velocity will result in large shear stress on the surface of the tumor. This shear stress can influence the cell function, proliferation and differentiation [32]. Hence, in studying cancer cells in a microfluidic device, the shear stress must not exceed a certain limit so that the culture condition agrees with in vivo conditions. According to Shemesh et al. [33], the shear rate in the range of 0.25–3 dyn/cm^2^ is appropriate for cancer cell culture under continuous flow. Therefore, the shear stress throughout this study is in a suitable range for cancer cell culture.

**Figure 6:**
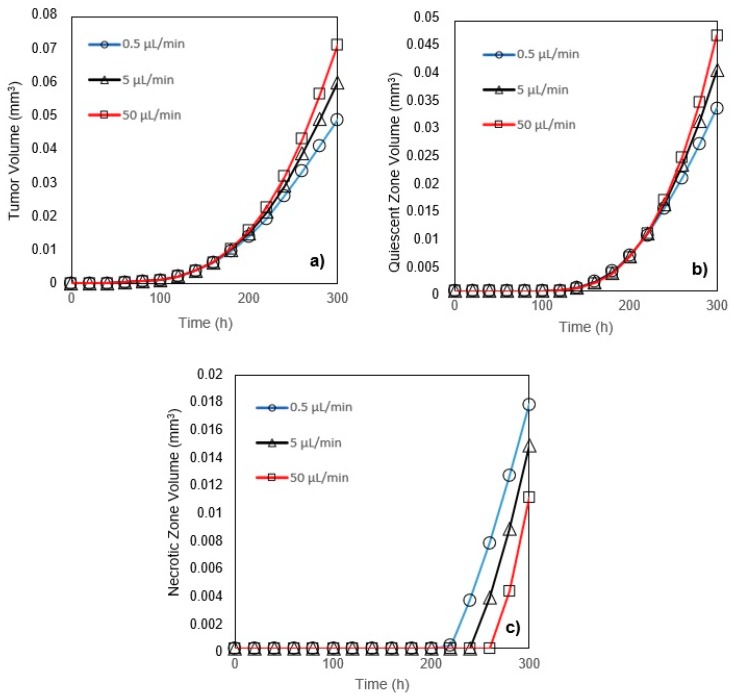
(**a**) Tumor volume; (**b**) Quiescent zone volume; (**c**) Necrotic zone volume for three inlet flows (0.5, 5, and 50 µL/min) versus time (glucose and oxygen concentration at the inlet are respectively 0.8 mM and 0.28 mM). The initial radius of the tumors in both of the traps is 24.3 μm.

A tumor grows more quickly when the inlet velocity increases since the nutrient delivery to the tumor is easier for the high-velocity inlet flow (Figure 6a). However, for all three inflow rates, while all tumors are in the first phase of their growth, their volumes at any instant are identical. The volume of the quiescent zone is larger for a tumor exposed to high-velocity inlet flow (Figure 6b). When compared to a low-velocity inlet, however, the volume of the necrotic zone is smaller in a tumor exposed to a high-velocity inflow (Figure 6c).

### 3.3. Comparison between Tumor Growth in Microchannels with U-Shaped Barrier and Microwell Trap 

**Figure 7:**
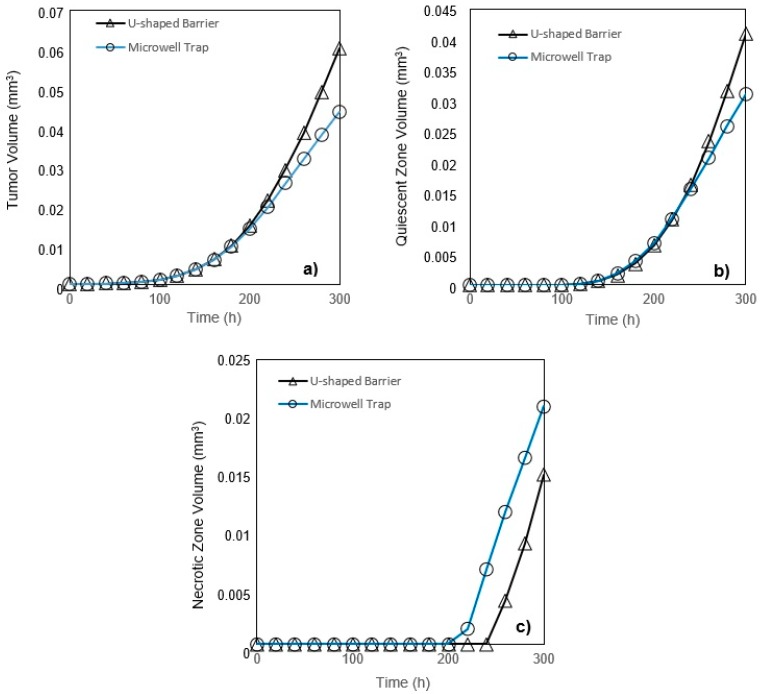
(**a**) Tumor volume; (**b**) Quiescent zone volume; (**c**) Necrotic zone volume for U-shaped barrier and microwell trap versus time (glucose and oxygen concentration at the inlet of each case are respectively 0.8 mM and 0.28 mM). The initial radius of the tumors in both of the traps is 24.3 μm.

In a microchannel with a U-shaped barrier, the tumor is directly exposed to the culture medium flow. However, in a microchannel with a microwell trap (Figure 2), nutrients do not reach the tumor surface as easily as they do in U-shaped barrier case and their velocity is considerably reduced when entering the microwell. Accordingly, the nutrient delivery to a tumor placed in a microwell trap is more complicated than the one in a U-shaped barrier. These results are completely compatible with the findings of Barisam et al. [19,27]. Generally, a tumor placed in a U-shaped barrier will grow faster than one in a microwell trap, but while both tumors are in the first phase of their growth, they will have the same volume (Figure 7a). At any instant after the first phase of the growth, quiescent zone volume is larger in a tumor in a microchannel with a U-shaped barrier (Figure 7b). In contrast, in the third phase of growth, the volume of the necrotic zone in the tumor in a microwell trap is larger (Figure 7c). 

**Figure 8:**
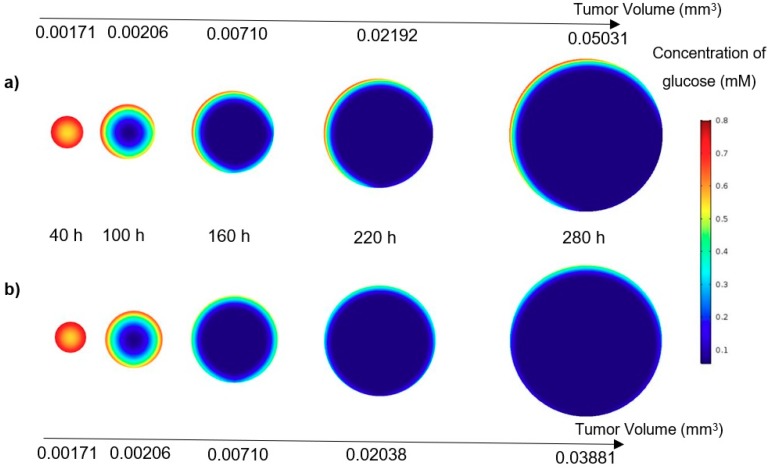

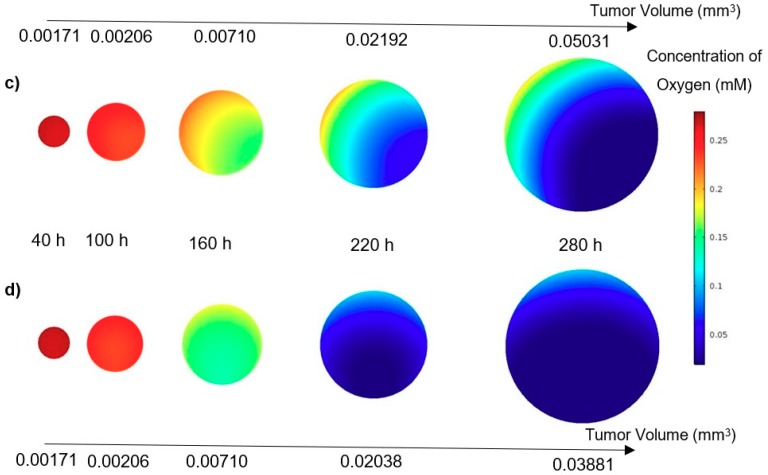
(**a**) Glucose concentration in a tumor in the U-shaped barrier; (**b**) Glucose concentration in a tumor in the microwell trap; (**c**) Oxygen concentration in a tumor in the U-shaped barrier; (**d**) Oxygen concentration in a tumor in the microwell trap versus time (concentrations of the glucose and oxygen in the inflow of culture medium are respectively 0.8 mM and 0.28 mM). All the contours are depicted in the XZ plane (see Figure 2).

Subsequently, as a graphical analogy, the contours of nutrient concentrations for a tumor in the U-shaped barrier and microwell trap, all in the plane XZ, are shown in Figure 8. From both contours of oxygen and glucose, it can be observed that the concentrations of the nutrients are higher in the tumor placed in U-shaped barrier trap, especially in the later stages of the growth. Moreover, due to the geometry of the traps, the zones lacking nutrients develop from the bottom for the tumor in the microwell trap and down-right corner for the one in the U-shaped barrier. The nutrient concentrations of the inflow for both cases are the same—0.28 mM oxygen and 0.8 mM glucose. Also, the volumetric flow rate of the inflow is 5 μL/min for both cases. 

## 4. Limitations and Suggestions

In the present study, the effect of extracellular matrix on tumor growth has not been considered. Actually spheroids in this examination are matrix-free. Certainly, the presence of extracellular matrix affects the distribution of oxygen and glucose [34,35] by changing the rate of diffusion and consumption. It is also a physical barrier to cell growth [36]. This model can be developed to be used for such problem if the information on nutrients diffusion and consumption and growth parameters under this condition is available.

Another limitation is that only one cell type was examined in this study because the mentioned information on the other cell types is very limited. Therefore, experimental studies are necessary to obtain the required parameters for the simulation.

Tumor growth is divided into two stages, avascular and vascular growth [37]. The present study, considering that it merely focused on avascular tumor growth, is only able to be used as a tool in designing drug therapies for patients with this stage of the disease. To this end, the drug concentration distribution equation in the tumor and the effect of drug concentration on the growth of cancer cells need to be added to the model. Such a model can also be used in combination with angiogenesis models to evaluate vascular tumor growth. It also can be utilized to study the growth of multicellular spheroids in other microfluidic systems containing concentration gradient generators and a variety of cell traps.

## 5. Conclusions

In this study, a modified mathematical model for tumor growth was first presented. In the modified model, it was considered that cells in the quiescent zone consume nutrients at the half rate of the proliferative zone consumption rate. Using the model for static, unlimited culture medium, the model was validated with the experimental data in the literature and was in an excellent agreement. By inspecting the effect of the nutrient concentrations on the growth of a tumor exposed to the static, unlimited culture medium, it was concluded that the determinative nutrient in retarding the tumor growth was glucose and that the concentrations of glucose and oxygen within the tumor respectively specify the volume of the quiescent and necrotic zones. In addition, by studying the growths of the tumors with different initial radii in the static, unlimited culture medium, we observed that the initial tumor radius does not significantly affect the final volume of the tumor. Then, by employing the equations concerned with the culture medium flow, the model was applied to the 3D growth of the tumor in the microfluidic system. The tumor growth in a microfluidic device containing a U-shaped barrier for three flow rates of the culture medium was investigated. The tumors developed faster when the inflow rate was increased, and nutrient delivery to the tumor surface was more difficult in microchannels with low inflow rate. Subsequently, the tumor growth in a microfluidic device containing a microwell trap was investigated. The results revealed that in equal inflow rate, the tumor grows slower in the microwell trap in comparison to the U-shaped barrier. The results also showed that the reduced velocity of the nutrient delivery to the tumor surface in the microwell trap was the reason of its slower growth rate, in comparison with the one in the U-shaped barrier. The approach of this study can be useful for further research on the growth of other tumor types in microfluidic devices. Furthermore, the proposed model can be helpful for experimental investigations in the growth of avascular tumor growth.

## Figures and Tables

**Table 1:** The values of the parameters used in the proposed model.

Parameters	Values	Units	Descriptions
ρ	993.3	kg/$m^{3}$	Density of water at 37 °C, [28]
Μ	6.92$\;\times 10^{-4}$	Pa·s	Viscosity of water at 37 °C, [28]
$D_{glucose-water}$	9.27$\;\times 10^{-10}$	$m^{2}/s$	Diffusion coefficient of glucose through water [28]
$D_{O_{2}-water}$	2.6$\;\times 10^{-9}$	$m^{2}/s$	Diffusion coefficient of oxygen through water [28]
$D_{1}$	4.22$\;\times 10^{-11}$	$m^{2}/s$	Diffusion coefficient of glucose through EMT6/Ro cancerous tissue [26]
$D_{2}$	1.65$\;\times 10^{-9}$	$m^{2}/s$	Diffusion coefficient of oxygen through EMT6/Ro cancerous tissue [26]
$C_{glucose-critical}$	0.06	mM	Critical concentration of glucose for EMT6/Ro spheroid [26]
$C_{O_{2}-critical}$	0.02	mM	Critical concentration of oxygen for EMT6/Ro spheroid [26]
$V_{1}$	4.36$\;\times 10^{-2}$	mol/$m^{3}/s$	Maximum consumption rate of glucose for EMT6/Ro spheroid [29]
$V_{2}$	2.74$\;\times 10^{-2}$	mol/$m^{3}/s$	Maximum consumption rate of oxygen for EMT6/Ro spheroid [29]
$K_{1}$	4$\;\times 10^{-2}$	mol/$m^{3}$	Michaelis constant of glucose for EMT6/Ro spheroid [28]
$K_{2}$	4.64$\;\times 10^{-3}$	mol/$m^{3}$	Michaelis constant of oxygen for EMT6/Ro spheroid, Casciari et al. [12]
$A_{p}$	1.2153$\;\times 10^{-9}$	1/s	Apoptosis rate constant for EMT6/Ro spheroid, Mahmood et al. [30]
$A_{n}$	2$\;\times 10^{-6}$	1/s	Necrosis rate constant for EMT6/Ro spheroid, Mahmood et al. [30]
A	$3.3\times 10^{-2}$ *	1/h	Proliferation rate constant for 0.8 mM glucose and 0.28 mM oxygen for EMT6/Ro spheroid [14]

* Obtained from the exponential cell doubling time (21 h).
